# Asymmetric Phenyl Substitution: An Effective Strategy to Enhance the Photosensitizing Potential of Curcuminoids

**DOI:** 10.3390/ph15070843

**Published:** 2022-07-09

**Authors:** Guglielmo Vesco, Martino Brambati, Luca Scapinello, Andrea Penoni, Massimo Mella, Màr Masson, Vivek Gaware, Angelo Maspero, Luca Nardo

**Affiliations:** 1 Department of Science and High Technology, Università degli Studi dell’Insubria, Via Valleggio 11, 22100 Como, Italy; gvesco@uninsubria.it (G.V.); mbrambati1@studenti.uninsubria.it (M.B.); l.scapinello@uninsubria.it (L.S.); andrea.penoni@uninsubria.it (A.P.); massimo.mella@uninsubria.it (M.M.); 2 School of Health Sciences, University of Iceland, Saemundargata 2, 102 Reykjavìk, Iceland; mmasson@hi.is (M.M.); vivekgaware@gmail.com (V.G.)

**Keywords:** curcuminoid, photosensitizer, excited-state intramolecular proton transfer, keto–enolic semi-aromatic ring, UV-Vis absorption spectroscopy, fluorescence, nuclear magnetic resonance, electronic structure calculations, singlet oxygen quantum yield

## Abstract

Curcumin has been demonstrated to exhibit photosensitized bactericidal activity. However, the full exploitation of curcumin as a photo-pharmaceutical active principle is hindered by fast deactivation of the excited state through the transfer of the enol proton to the keto oxygen. Introducing an asymmetry in the molecular structure through acting on the phenyl substituents is expected to be a valuable strategy to impair this undesired de-excitation mechanism competing with the therapeutically relevant ones. In this study, two asymmetric curcumin analogs were synthesized and characterized as to their electronic-state transition spectroscopic properties. Fluorescence decay distributions were also reconstructed. Their analysis confirmed the substantial stabilization of the fluorescent state with respect to the parent compound. Nuclear magnetic resonance experiments were performed with the aim of determining the structural features of the keto–enol ring and the strength of the keto–enol hydrogen bond. Electronic structure calculations were also undertaken to elucidate the effects of substitution on the features of the keto–enol semi-aromatic system and the proneness to proton transfer. Finally, their singlet oxygen-generation efficiency was compared to that of curcumin through the 9,10-dimethylanthracene fluorescent assay.

## 1. Introduction

Curcumin, **1**, the yellow-orange pigment extracted from the rhizome of curcuma longa, is known for its multiple biological and pharmaceutical properties [1,2,3,4,5,6]. It has been recognized to have anti-inflammatory and healing potency since the ancient Indian medicine textbooks and it is cited in the Ayurveda [7,8]. In the last 30 years, it has been the subject of a huge number of scientific studies [9,10,11,12], and turned out to have unique pharmaceutical potential due to its ample spectrum of biological activities, spanning from chemopreventive [13,14] and chemotherapeutic effects [1,14,15,16], to anti-Alzheimer [17,18] and anti-AIDS [19,20] properties. Although being edible in high doses when it is in its ground state (it is the main constituent of the spice Curcuma, as well as of curry, and is used in the alimentary industry as yellow dye) [21], since the end of the XX century it has been observed that **1** displays phototoxic properties [22,23,24]. Lately, its potential as a photosensitized bactericidal has been demonstrated and tested within different formulations and on several Gram-positive and negative bacteria [25,26,27]. Moreover, thanks to the massive work undertaken by several research groups in order to devise formulations capable of delivering **1**, which is only slightly soluble in water at mildly acidic pH and prone to severe hydrolysis at physiological and basic ones [28,29], to biological tissues [30,31,32,33,34,35], the out-of-lab application of this active principle in clinical practice is now conceivable.

On the other hand, the full exploitation of **1** as a photosensitizer is hindered by the occurrence of a plethora of deactivation mechanisms involving, as a common feature, the transfer of the enol proton to the keto oxygen in the first excited singlet state, S_1_, with concomitant dissipation of the excitation energy to overcome the excited-state intramolecular proton transfer (ESIPT) reaction activation potential barrier and relaxation to the ground state [36,37,38,39,40,41,42,43,44]. Although the exact mechanisms by which **1** explicates its photosensitized activity are not fully understood yet [45,46], the stabilization of S_1_ would, in any case, enhance the probability for any phototoxic reaction to take place. The stability of the S_1_ state and the excited-state dynamics of a fluorophore can be probed through the measurement of its time-resolved fluorescence decay distribution [47,48]. In the recent past, **1** was the object of several in-depth time-resolved fluorescence studies [36,38,49,50], which revealed that the S_1_ average fluorescence lifetime is in the range of tens to hundreds of picoseconds in a wide range of organic solvents differing in polarity and hydrogen-bonding properties, with the main non-radiative decay mechanism being ESIPT and the rate-limiting step of this mechanism being the rearrangement of solvent molecules that partially quench keto–enol intramolecular hydrogen bonding (KEIHB). During the last decade, our group has been engaged in the characterization of the excited-state dynamics and reactive oxygen species (ROS)-generation efficiency of several naturally occurring as well as synthetic curcumin analogs [39,40,41,42,43,44]. All of the above compounds shared a symmetric structure with respect to the vinylic carbon, and all of them exhibited strong KEIHB and thus fast ESIPT. Extensive resonance among the single and double C–C bonds of the molecular heptadiene backbone is required for the ESIPT reaction to take place. Thus, introducing an asymmetry in the molecular structure through acting on the phenyl substituents is expected to be a valuable strategy to hinder this undesired de-excitation mechanism competing with therapeutically relevant ones.

**Figure 1:**
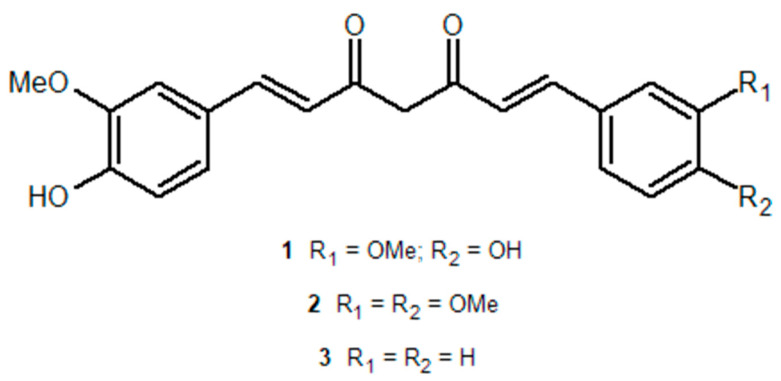
Chemical structures of the investigated curcuminoids.

In the present study, the two asymmetric curcumin analogs **2** and **3** (see Figure 1) were synthesized according to previously published procedures [51] and characterized as to their electronic-state transition spectroscopic properties. Namely, UV-Vis absorption as well as fluorescence excitation and emission spectra were recorded. Fluorescence decay distributions were also reconstructed with 30 picoseconds temporal resolution by applying the time-correlated single-photon counting (TCSPC) technique. The analysis of such decays confirmed a substantial stabilization of the fluorescent state with respect to the parent compound **1** in inert solvents. This evidence encourages further synthetic efforts to devise asymmetric curcuminoids whose excited state is not quenched by solute–solvent interactions in the excited state, even in more reactive environments. Nuclear magnetic resonance (NMR) experiments were performed with the aim of determining the structural features of the keto–enol ring and the effects of solvent on the KEIHB strength. Electronic structure calculations were also undertaken to elucidate the effects of substitution on the features of the keto–enol semi-aromatic system and the proneness to proton transfer. Finally, the relative efficacy of **2** and **3** compared to curcumin in photosensitizing the production of ROS in vitro was preliminary assessed through the 9,10-dimethylanthracene (DMA) fluorescent assay.

## 2. Results and Discussion

### 2.1. Electronic-State Transition Spectroscopic Properties of Compound **2**

The spectral properties of **2** are reported hereafter. The same studies performed on compound **3** are detailed in Section 2.2.

#### 2.1.1. UV-Vis Absorption

The absorption spectra of **2** were acquired in all of the solvents of Table 1. The absorption spectrum consists of a single broad band peaking at around 420 nm, with a shoulder at longer wavelengths. This suggests, by comparison with **1** as well as other curcuminoids and β-diketones [36,37,38,39,40,41,42,43,44,52], that the keto–enol equilibrium is notably shifted towards the enol tautomers, with the diketo tautomers appearing at most in traces.

**Figure 2:**
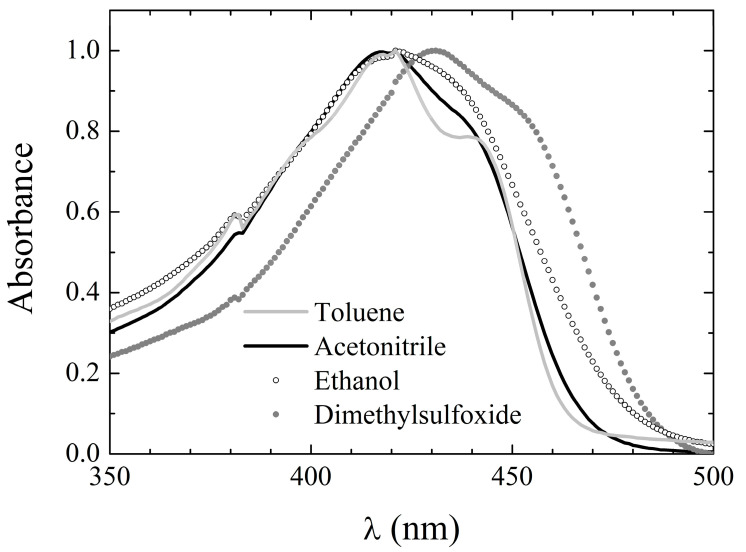
Absorption spectral line shape of **2** in exemplary solvents.

The NMR spectroscopy and in silico calculations support the above conclusion (vide infra). The absorption peak wavelength in the various solvents is reported in Table 2, while exemplary spectra in selected solvents are shown in Figure 2. The spectral features of **2** appear to be only slightly dependent on the environment, although a modest red shift and a loss in structure is observed by changing from inert (cyclohexane and toluene) to reactive environments. This behavior partially contrasts with that of **1 [38]**, for which the benchmarks of the environmental properties were more evident in the spectral features. Indeed, an additional shift to longer wavelengths was observed in the absorption peak by dissolving the compound in H-bonding compared to non-H-bonding solvents. The absorption peaks of **1** in the different solvents are reported beneath the ones of **2** in Table 2 for the sake of straightforward comparison.

The molar extinction coefficient at the absorption peak was determined for **2** as detailed in the Materials and Methods section in all solvents excluding cyclohexane and ethylene glycol, where the low solubility of **2** forced us to filter the sample prior to spectrum acquisition in order to avoid severe scattering. It is in the range of 30,000 M^−1^cm^−1^ and does not appear to depend systematically on the solvent properties.

#### 2.1.2. Steady-State Fluorescence

**Figure 3:**
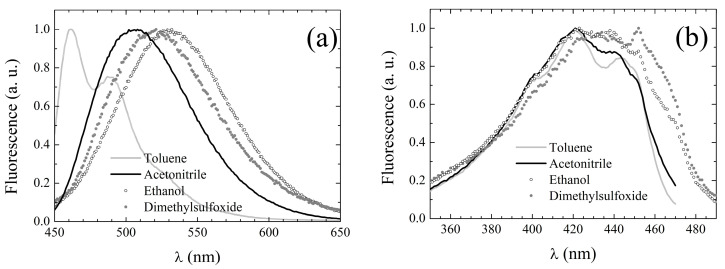
Fluorescence emission (**a**) and excitation (**b**) spectral line shapes of **2** in exemplary solvents.

The fluorescence emission spectra of **2** are broad and structureless, with the exception of those acquired in non-polar solvents in which two distinct bands can be resolved. In toluene, the spectrum exhibits two peaks, while in cyclohexane, the longer-wavelength band appears as a shoulder. The peak emission wavelengths are reported in Table 3, while exemplary spectra in selected solvents can be inspected in Figure 3a. Unlike the absorption spectra, the emission spectra appear to be reminiscent of the solvent properties. However, like for **1**, the dependence is not trivially amenable to bare polarity effects. Indeed, in H-bonding solvents (both H-bond acceptors and alcohols), fluorescence is systematically red-shifted with respect to polar weakly H-bonding solvents of comparable polarity. Nonetheless, the solvent-induced red-shift is much reduced for **2** with respect to the parent compound (whose emission peak wavelengths are also reported in Table 3 for the sake of straightforward comparison), particularly in H-bonding solvents. The most striking difference between **1** and **2** is constituted by the notably higher fluorescence quantum yield of the latter (see fourth column in Table 3) with respect to the former (fifth column). The fluorescence quantum yield of **2** could not be reliably assessed in cyclohexane and ethylene glycol due to the necessity of working on very diluted samples after filtering the solution.

Fluorescence excitation spectra were also recorded, setting the observation wavelength at the emission peak whenever it was >500 nm, at 500 nm otherwise. The excitation peak wavelengths are listed in Table 3, column 6, and appear to be only slightly red-shifted with respect to the corresponding absorption peak wavelengths (see Table 2). The excitation spectral line shapes also conserve the main features of the corresponding UV-Vis absorption spectra, as evidenced by the exemplary spectra displayed in Figure 3b. These features suggest that S_1_ deactivation simply occurs through inverse transition backwards to the ground state with no involvement of additional electronic energy levels.

#### 2.1.3. Excited-State Dynamics

The best-fitting parameters retrieved from the fit of the experimental decay distributions measured for **2** in the different solvents are reported in Table 4. The values of the average S_1_ state lifetime, defined as

(1) $$\tau_{av}=\sum_{i}\tau_{i}f_{i},$$

where the *τ_i_* are the decay times resolved within the decay pattern with fractional amplitudes *f_i_*, and the summation is performed over all the detected transients, are also listed in the fifth column.

The fluorescence decay of **2** is essentially single-exponential in all of the tested solvents, with the exceptions of cyclohexane and methanol. This behavior contrasts with that observed for **1**, which exhibits two exponential decays in all H-bonding solvents [38]. In the frame of the model proposed in [38] and consolidated by the analysis of several other symmetric curcumin analogs [39,40,41,42,43,44], the non-radiative decay mechanisms taking place for **1** are:

- Direct ESIPT (occurring in non-polar solvents),
- Reketonization (occurring in non-polar solvents and triggered by the lower polarity of the trans-diketo with respect to the enol tautomers),
- Solvent rearrangement-moderated ESIPT (occurring in polar solvents, H-bonding or not),
- Intermolecular charge/energy transfer with H-bonding solvent molecules.

By analogy, we can try to interpret the excited-state dynamics of **2** in terms of the same mechanisms. It turns out that direct ESIPT occurs only in cyclohexane, with a decay time similar to that measured for **1**, but with much lower probability. Concomitantly, reketonization occurs on timescales very similar to those measured for **1**, but with higher probability. The same considerations also hold true in the second non-polar solvent we tested, toluene, in which **1** was not previously characterized. Its decay is two-exponential, with time constants *τ*_1_ < 30 ps and *τ*_2_ = (332 ± 2) ps, and relative amplitude of the shortest component f_1_ = 0.72 ± 0.02, while for compound **2**, the shorter decay component is not even resolved, suggesting that direct ESIPT takes place with negligible probability and the main decay mechanism is reketonization. The net effect is a two-fold increase in the average S_1_-state lifetime in non-polar environments (the average lifetime for **1** is reported in the last column of Table 4 for the sake of straightforward comparison).

In polar, non-H-bonding solvents, the decays are single-exponential, suggesting that the excited-state deactivation occurs through pathways qualitatively similar to those experienced by the parent compound **1** as well as by other previously investigated symmetric curcuminoids [38,39,40,41,42,43,44], with the driving mechanism being solvent rearrangement-moderated ESIPT. The excited-state lifetimes in the different solvents are comparable to, although typically somewhat shorter than, those measured for **1**.

In H-bonding solvents, for **1**, non-radiative decay through intermolecular charge/energy transfer with the solvent was predominant, but solvent rearrangement-moderated ESIPT still occurred with non-negligible probability. For **2**, we assisted a significant enhancement of the relative probability of the former at the expense of the latter mechanism, with the overall effect of only slightly stabilizing the excited state in spite of the fact that both decay mechanisms are systematically slower in all of the probed solvents for the asymmetric than for the parent compound.

The excited-state dynamics can be further investigated by estimating the values of the radiative decay rate constant, *k_fl_*, and the non-radiative decay rate constant, *k_nr_*, from the measured *τ_av_* and *ϕ_fluo_*, according to the following relations:

(2) $$k_{fl}=\frac{\phi_{fluo}}{\tau_{av}}$$

(3) $$k_{nr}=\frac{1}{\tau_{av}}-k_{fl}$$

The calculated values of *k_fl_* and *k_nr_* in the tested solvents are reported in Table 5 (columns two and four, respectively) and compared with those calculated for **1** (columns three and five, respectively) [38]. The non-radiative rate constant is systematically lower for **2** than for **1** in all of the solvents except butanol. However, concomitantly, fluorescence photons are emitted at a higher rate by the former compound.

### 2.2. Electronic-State Transition Spectroscopic Properties of Compound **3**

#### 2.2.1. UV-Vis Absorption

**Figure 4:**
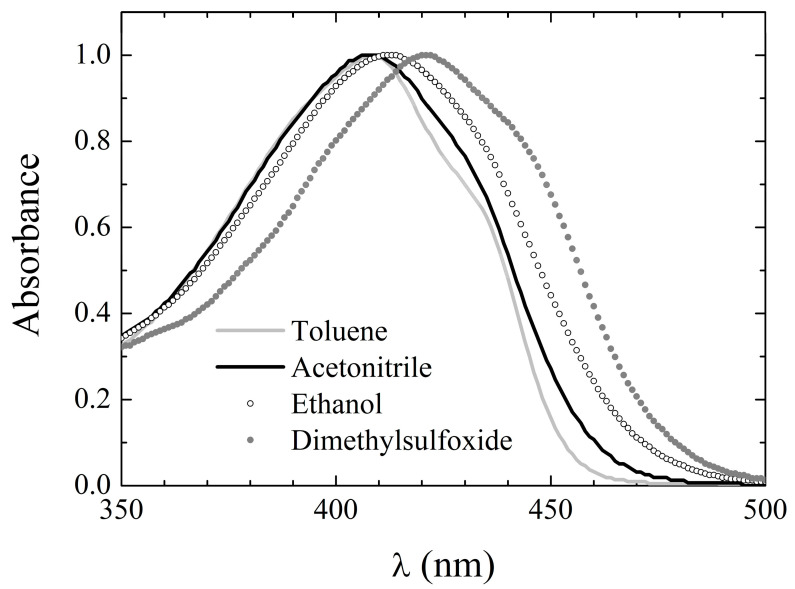
Absorption spectral line shape of **3** in exemplary solvents.

The absorption spectra of **3** were acquired in all of the solvents of Table 1. The absorption spectrum consists of a single broad band peaking at around 410 nm, occasionally with a shoulder at longer wavelengths. Thus, also in the case of **3**, the keto–enol equilibrium seems to be shifted towards the enol tautomers, with the diketo tautomers appearing at most in traces, as confirmed by both the NMR and in silico calculations (vide infra). The absorption peak wavelength in the various solvents is reported in Table 6, while exemplary spectra in selected solvents are shown in Figure 4. The absorption bands of **3** are slightly, though systematically, blue-shifted with respect to those of both **1** and **2**, which might indicate a reduced charge conjugation in **3**. Interestingly, the same observation pertained to dicinnamoylmethane (DCMeth), a symmetric, non-phenyl substituted curcuminoid [39], whose absorption peak wavelength is reported as λ_abs,DCMeth_ in the third column of Table 6.

Moreover, the spectral features of **3** are less dependent on the environment than those of both **1** and **2**, although a loss in structure can be still observed by changing from inert (cyclohexane and toluene) to reactive environments. This behavior is, again, similar to the one exhibited by DCMeth [39].

The molar extinction coefficient of **3** in all tested solvents is in the range of 30,000 M^−1^cm^−1^ and does not appear to depend systematically on the solvent properties. Moreover, it is slightly, but systematically, lower than that of **2**, stemming from further support of a reduced charge conjugation in **3**.

#### 2.2.2. Steady-State Fluorescence

**Figure 5:**
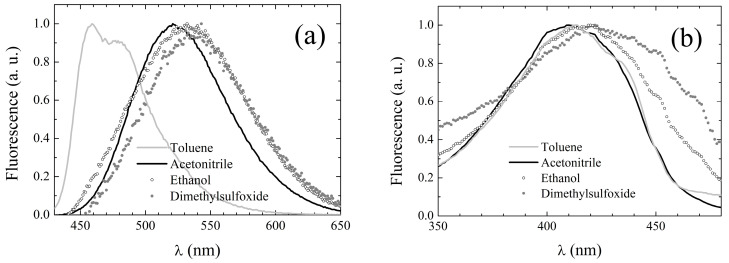
Fluorescence emission (**a**) and excitation (**b**) spectral line shapes of **3** in exemplary solvents.

The peak fluorescence emission wavelengths of **3** in the different solvents of Table 1, obtained upon excitation at the pertaining λ_abs_, are reported in Table 7, while exemplary spectra in selected solvents can be inspected in Figure 5a.

Unlike the absorption spectra, the emission spectra appear to resemble those of **1** rather than of DCMeth. First, the spectra of **3** are broad and structureless, with the exception of those acquired in non-polar solvents. In cyclohexane, the spectrum exhibits two peaks with a further shoulder at long wavelengths, while in toluene, the spectrum consists of a main peak and a red-shifted shoulder. Conversely, those of DCMeth were double-peaked in all of the tested solvents (see third column of Table 7). Moreover, the fingerprints of the solvent dependence of the fluorescence emission typical of **1** are also exhibited by **3**, even more neatly than by **2**. Although a direct correlation between peak emission wavelength and polarity is not observed, in the polar solvents, the emission spectrum is generally peaked at longer wavelengths than in inert ones. Furthermore, in the H-bonding solvents (both H-bond acceptors and alcohols), the fluorescence is systematically red-shifted with respect to polar weakly H-bonding solvents of comparable polarity. The fluorescence quantum yield of **3** (see fourth column in Table 3) is notably higher than that of **1** in non-polar (cyclohexane and toluene) and slightly polar (chloroform and dichloromethane) solvents, while it is comparable in highly polar environments and sizably lower in H-bonding solvents with the only exception of ethylene glycol. The comparison with **2** unravels a systematic reduction in the quantum yield for **3** with respect to the other asymmetric compounds, which might correlate with the specific phenolic substituents. Indeed, DCMeth was characterized by a particularly low quantum yield in all solvents [39] (the values are reported in the fifth line of Table 7 for the sake of straightforward comparison).

Fluorescence excitation spectra were also recorded, setting the observation wavelength at the emission peak whenever it was >500 nm, and at 500 nm otherwise. The excitation peak wavelengths are listed in Table 7, column 6, and appear to be only slightly red-shifted with respect to the corresponding absorption peak wavelengths (see Table 6). Moreover, the excitation spectral line shapes conserve the main features of the corresponding UV-Vis absorption spectra, as evidenced by the exemplary spectra displayed in Figure 5b. These features suggest that for **3**, S_1_ deactivation also simply occurs through inverse transition backwards to the ground state with no involvement of additional electronic energy levels.

#### 2.2.3. Excited-State Dynamics

The best-fitting parameters retrieved from the fit of the experimental decay distributions measured for **3** in the different solvents are reported in Table 8. The values of the average S_1_ state lifetime, defined according to Equation (1), are also listed in the fifth column.

In inert environments, a behavior similar to that of **2** is observed, with **3** displaying a three-exponential decay pattern in cyclohexane and an essentially single-exponential decay in toluene. However, in cyclohexane, the ESIPT rate appears to be much faster for **3** than for both **1** and **2**, and is comparable with that measured in DCMeth. Moreover, the relative probability of **3** to decay by means of direct ESIPT is comparable with that measured for **1**, and is much higher than that of **2**. Reketonization occurs on timescales similar to those typical of both **1** and **2**, and faster than for DCMeth.

The net effect is that the overall stability of the excited state of **3** is comparable to that of **1** and inferior to that of **2** (see the fifth column of Table 8 and the last column of Table 4 for the average lifetimes of **3** and **1**, respectively). Conversely, in toluene, like for **2**, the main decay transient has the time constant typical of reketonization dynamics. The effect is a two-fold increase in the average S_1_-state lifetime of **3** with respect to **1** in this solvent. In both non-polar solvents, the presence of traces of the non-polar trans-diketo isomer is denounced by the longest-lived component.

In polar, weakly H-bonding solvents, **3** seems to exhibit a behavior similar to that of **1**. The decay patterns are essentially single-exponential, suggesting that solvent rearrangement-moderated ESIPT is the main decay mechanism. This latter occurs systematically on faster timescales with respect to both **1** and **2**. The residual, long-lived component resolved in the weakly polar solvents chloroform and dichloromethane (ε < 10) can be ascribed to traces of anti-diketo isomer, which were also observed in DCMeth in solvents of comparable polarity. The survival of traces of **3** in the trans-diketo isomer even in mildly polar environments agrees with a reduced polarity difference between the latter and the enol tautomers with respect to **1** and **2**.

The decays of **3** are multi-exponential in most of the other solvents, revealing complex and hardly interpretable deactivation photophysics. In H-bond-accepting solvents, benchmarks of the DCMeth excited-state dynamics are recognizable in the decays of **3**. Indeed, for both **3** and DCMeth, the fluorescence decay patterns in DMF and DMSO are dominated by a transient with time constant in the range typical of direct ESIP mechanisms. Some valuable hints can be extracted from this analogy. Indeed, in the case of DCMeth, the enhanced proneness to decay by direct ESIPT in any environment was ascribed to the formation of a particular variant of the closed enol tautomer having c_2ν_ symmetry, in which the enol proton resulted in being delocalized and equally shared between the two keto oxygens, together with the closed enol structure with the enol proton localized in proximity to either one of the two keto oxygens adopted by **1** and several other curcuminoids. In particular, because in the c_2ν_ structure the enol proton is more acidic, the latter is expected to be dominant in H-bond-accepting solvents, where a transient transfer of the enol proton to the H-bond-accepting moieties might be responsible for fostering the exchange of the proton itself between the two keto oxygens. This mechanism was invoked in [39] to explain the very complex fluorescence decay distributions measured for DCMeth. The observation of a transient typical of direct ESIPT in the decay of **3** in H-bond-accepting solvents suggests that, as in DCMeth, the keto–enolic ring is more likely to assume the symmetric c_2ν_ structure than in **1** and **2**. Moreover, explicit-solvent simulations on **3**’s interactions with DMF unraveled a sufficiently strong affinity between the enol proton of **3** and the solvent carbonylic oxygen (with an equilibrium distance of 2.43 Å) to distort the hydrogen-bonding structure, reducing the enolic O–H distance by 0.03 Å and increasing the distance between the proton and the ketonic oxygen by 0.22 Å. In our view, this finding suggests a reduced likelihood for the direct ESIPT and prefigures the transient solute–solvent proton transfer described above as being likely to take place for **3** as well. A second transient accounts for roughly 1/3 of the excited-state deactivation events and has a lifetime in the range of a few hundred picoseconds, typical of the solute/solvent charge/energy transfer dynamics observed in H-bonding solvents for DCMeth, **1**, and **2**. Finally, a third component is barely detectable, which we ascribe to decay through solvent rearrangement-moderated ESIPT. The latter is similar, in time and amplitude, to the homologous contributions measured in the decays of DCMeth and **2** in H-bond acceptors, while the same deactivation mechanism occurs on significantly shorter timescales for **1**, suggesting the formation of a looser intermolecular H-bond between the solvents’ H-bond-accepting moieties and the enol proton in the parent compound.

In alcohols, the decay dynamics seem to depend on the specific solute/solvent interactions. Indeed, although the intermolecular charge/energy transfer dynamics occur in all alcohols on timescales similar to those observed for this mechanism in **1**, **2**, and DCMeth, as well as for **3** itself in H-bond acceptors, an additional decay component is resolved in methanol and ethylene glycol, whose time constant is in the range typical of direct ESIPT, which was observed in all alcoholic solvents for DCMeth. This suggests that, in the case of these solvents, a sort of bridging interaction between the enol proton, the H-bond-accepting moiety of the alcohol, the protic moiety, and the unshielded keto oxygen might be established. Interestingly, these solvents are those endowed with the highest Kamlet–Taft acidity parameters among those used in this study. Moreover, the bridging effect seems to be most effective in promoting the enol proton transfer in methanol, which is the smallest solvent and is thus more likely to juxtapose between the enol and keto moiety of **3**.

The overall effect of these complex excited-state dynamics is to destabilize the excited state of **3** with respect of **2** and even of **1**. However, the electron-withdrawing character of non-substituted phenyl rings in the molecular structure of a curcuminoid was postulated to have a highly destabilizing effect on the excited state [53] through favoring ESIPT dynamics. In this respect, the introduction of asymmetry in the molecular structure produces a consistent stabilization of **3** compared to DCMeth, the most unstable curcuminoid we have examined until now, which bears non-substituted phenyl rings at both sides of the keto–enol system, and whose average excited-state lifetimes are reported in the last column of Table 8 for the sake of straightforward comparison.

The excited-state dynamics can be further investigated by estimating the values of the radiative decay rate constant, *k_fl_*, and the non-radiative decay rate constant, *k_nr_*, from the measured *τ_av_* and *ϕ_fluo_*, according to Equations (2) and (3). The calculated values of *k_fl_* and *k_nr_* in the tested solvents are reported in Table 9 (columns two and four, respectively) and compared with those calculated for DCMeth (columns three and five, respectively) [39]. We recall that the homologous data for **1** are listed in Table 5, columns three and five. The non-radiative rate constant is systematically lower for **3** than for DCMeth in polar, weakly H-bonding solvents, where it is in the same order of magnitude as that of **1**, while in both inert and H-bonding environments, the tendency to decay through non-radiative pathways of **3** and DCMeth are comparable. In all of the solvents, the radiative decay rate of **3** is in the same order of magnitude of that of **1** and is much higher than that measured for DCMeth.

### 2.3. NMR Spectroscopy

**Figure 6:**
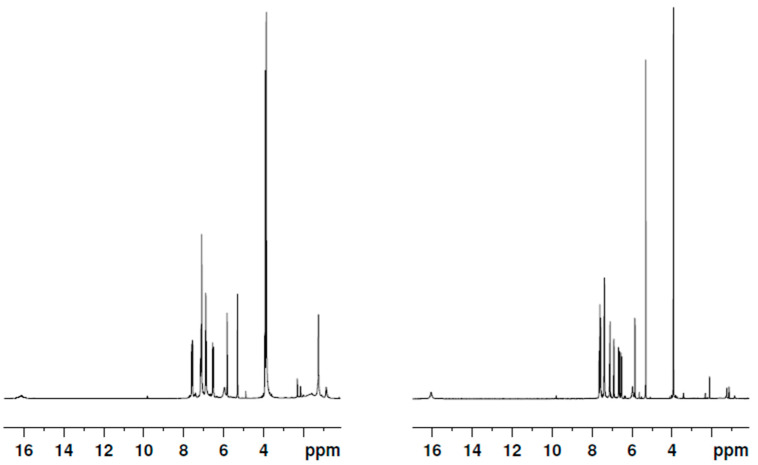
^1^H NMR spectra of **2** (**left**) and **3** (**right**) in dichloromethane-*d*_2_.

Curcuminoids **2** and **3** were characterized by ^1^H and ^13^C nuclear magnetic resonance spectroscopy in dichloromethane-*d*_2_ at room temperature. The ^1^H spectra are reported in Figure 6 and are compatible with those reported in [51]. For a complete attribution of the signals and their integration, please refer to the above-quoted reference. The compounds were further characterized in a panel of other solvents. The pertaining spectra are shown in Figure S1 (compound **2**) and Figure S2 (**3**) of the Supplementary Online Materials section.

**Figure 7:**
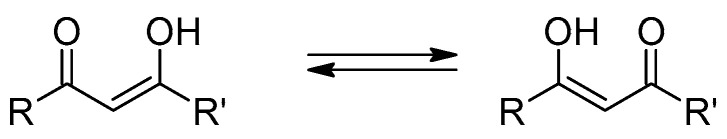
Keto–enol tautomer equilibrium.

As can be inferred by the spectra in Figure 6, Figures S1 and S2, and in agreement with the results reported in [51], in all of the inspected solvents, the keto–enolic equilibrium is totally shifted towards the enolic forms. We assumed the signal of the vinyl proton (in the region around 5–6 ppm chemical shift) as the benchmark of the enol tautomers and the signal of the methylene proton (around 3–4 ppm) as the benchmark of the diketo tautomers. This second peak was not observed in any of the spectra. Moreover, average resonance was detected due to the fast equilibrium between two equivalent asymmetric keto–enol tautomers (Figure 7), as demonstrated by the detection of a single vinyl proton peak in all spectra.

**Figure 8:**
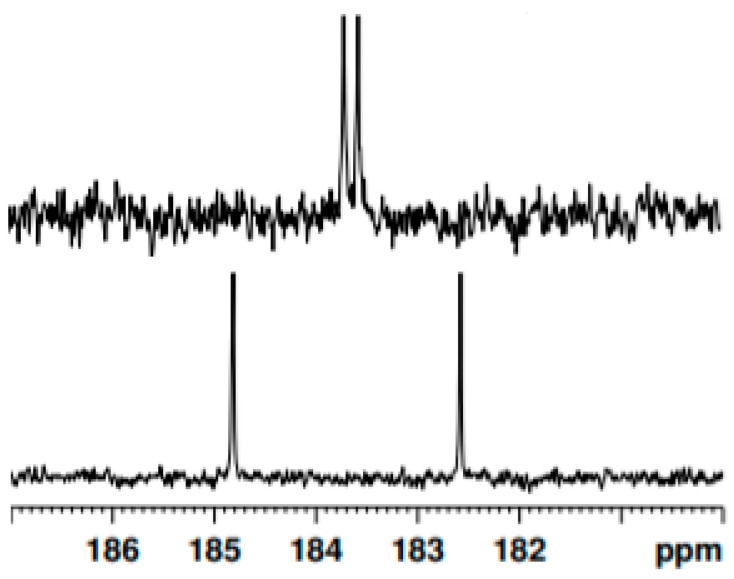
^13^C-NMR of species **2** (**up**) and species **3** (**down**) in dichloromethane-*d*_2_, which only shows quaternary carbon C-O.

The ^13^C-NMR spectrum of **2** and **3** clearly revealed two distinct resonances for quaternary C-O carbons, at 183.7 ppm and 183.5 ppm for species **2** and at 184.7 ppm and 182.5 ppm for species **3** (Figure 8). In principle, these double peaks might be explained by hypothesizing the concomitant presence of enol and diketo tautomers. However, as discussed above, the analysis of the ^1^H-NMR spectra showed that the keto–enolic equilibrium is totally shifted towards the enol tautomers. Alternatively, the two peaks of Figure 8 could be attributed to the signals of the carbons next to the protonated and non-protonated oxygen, respectively. Since such signals may only be resolved in the case of slow proton-transfer kinetics, this latter hypothesis can also be ruled out by the inspection of the ^1^H-NMR spectra, demonstrating that the enol proton is fluxional. Thus, the signals can be uniquely attributed to the two chemically inequivalent quaternary C–O carbons, confirming the asymmetric structure of the isolated curcuminoids. In contrast, the ^13^C-NMR spectrum of species **1** only shows one resonance for quaternary carbon C–O at 183 ppm, as the presence of a *C*_2_ symmetry axis makes the carbonyls chemically equivalent [54].

Concerning the proton NMR experiments, the structural asymmetry of compounds **2** and **3** was confirmed as their spectra showed distinct signals associated with methoxy groups, thus revealing a neat differentiation between the phenyl rings in both compounds **2** and **3**.

**Figure 9:**
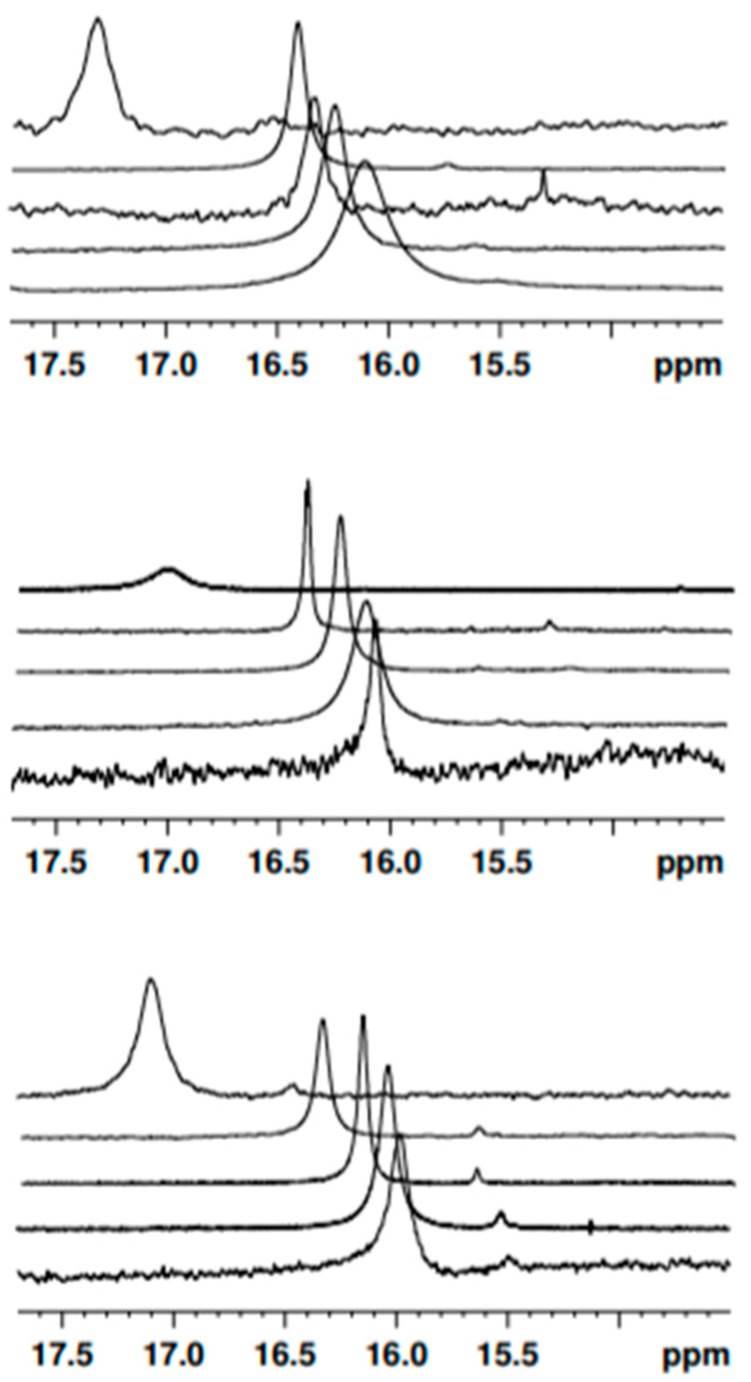
^1^H-NMR spectra of **1** (**up**), **2** (**center**), and **3** (**down**) in (from top to bottom) toluene-*d*_8_, dimethylsulfoxide-*d*_6_, acetonitrile-*d*_3_, dichloromethane-*d*_2_, and chloroform-*d*_1_.

Noticeably, in dichloromethane-*d*_2_, a signal for the species **1**, **2** and **3** can be detected as a broad band centered at 16.40 ppm, 16.21 ppm, and 16.10 ppm, respectively. This signal is originated by the enolic proton. Such a particularly low-field shift suggests its involvement in a keto–enol intramolecular hydrogen bond [55,56]. In fact, the characterization of KEIHB is a crucial step towards the development of curcuminoids endowed with excited-state dynamics optimized for applications in photodynamic therapy [44]. Once KEHIB was demonstrated in both **2** and **3**, we explored its structural features and strength in comparison to **1** in five different solvents (toluene-*d*_8_, dimethyl sulfoxide-*d*_6_, acetonitrile-*d*_3_, dichloromethane-*d*_2_, and chloroform-*d*_1_) by means of ^1^H-NMR (Figure 8). The ^1^H-NMR spectra recordings of compounds **1**, **2**, and **3**, in such a non-polar and non-hydrogen-bonding solvent as toluene-*d*_8_, show the enolic proton resonance at very low fields: 17.28 ppm, 17.04 ppm, and 17.10 ppm, respectively. This emphasizes particularly strong KEHIB. As a confirmation of the strength of the keto–enol intramolecular hydrogen bond, the ^1^H-NMR spectra recordings of curcuminoids **1**, **2**, and **3** in polar solvents, both non-hydrogen-bonding (dichloromethane-*d*_2_, chloroform-*d*_1_, and acetonitrile-*d*_3_) and H-bond-accepting (dimethyl sulfoxide-*d*_6_), revealed a rather small variation in the chemical shift of the enolic proton involved in KEHIB (Figure 9). The OH enolic proton chemical shift is related to the intensity of the hydrogen bond formed by the OH group. Small shifts of the resonance band associated with the enolic proton, evidenced in hydrogen-bond acceptors with respect to slightly polar solvents (less than 0.5 ppm towards higher fields), witness the high magnitude of the intramolecular hydrogen bond, which is only loosely perturbed by the interaction with the solvent itself.

In fact, ^1^H-NMR spectroscopy has already been used for hydrogen-bonding investigations [56,57]. In 2017, Abraham et al. provided an empirical formula to calculate hydrogen-bond acidity in solution [58,59]. Their parameter A is based on the OH and NH chemical shift (δ) difference between dimethyl sulfoxide-*d*_6_ and chloroform-*d*_1_ (A = 0.0065 + 0.133Δδ; Δδ = δ_OH enol_(DMSO) − δ_OH enol_(CDCl_3_)) [59], and can be used as a quantitative assessment of intramolecular hydrogen bonding. An A value for a characteristic OH group will give a quantitative estimate of the magnitude of KEHIB due to that OH. The parameter A was calculated for species **1**, **2**, and **3**, and was found to be less than 0.1, which confirms the presence of a very strong intramolecular hydrogen bond through a six-membered ring system (Table 10). However, it is worth noting that the strength of KEHIB scales inversely with the asymmetry character of the molecule, which confirms that the strategy of inducing asymmetries in the structural formula is indeed a valuable strategy to reduce undesired ESIPT mechanisms, thereby fostering the photosensitization of reactive oxygen species.

Although species **2** and **3** are characterized by an asymmetric structure, the relative ^1^H-NMR spectra at room temperature only shows one signal for the enolic proton due to the fast equilibrium between the two equivalent asymmetric keto–enol tautomers. In an attempt to extract information on the height of the potential barrier for proton transfer and estimating the transfer rates, we performed variable-temperature ^1^H-NMR studies in toluene and dichloromethane, reaching a low-limit temperature of 188 K. In Figures S3 and S4 of the Supplementary Online Materials section, we report the ^1^H-NMR spectra of **2** and **3**, respectively, in toluene at selected temperatures. In Figures S5 and S6, we focus on the enol proton peak and monitor its variations as a function of temperature. As can be evinced by the inspection of these plots, although the width and shift of the enol proton signal notably evolves as a function of temperature, even at the lowest temperature we could reach we did not manage to resolve distinct signals for the enol proton bound to either of the keto oxygens. Consequently, we were not able to obtain detailed kinetic information about the proton transfer. We can only state that the proton exchange between the two equivalent asymmetric keto–enol tautomers is fast on the NMR timescale, even at 188 K. The data acquired in dichloromethane were similarly elusive and are not shown.

### 2.4. Theoretical Modeling

**Figure 10:**
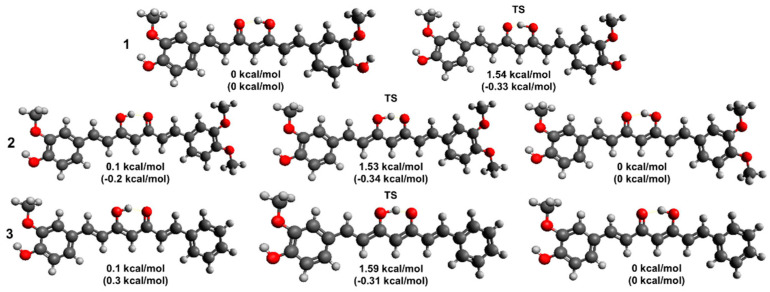
Optimized stationary point geometries and their relative energetics on the ground-state potential energy surface for the enolic tautomers of species **1**, **2**, and **3**, as obtained with the B3LYP/6-31++G(d, p) level of theory; also shown are the transition state structures (TS) for the proton transfer between the two keto–enolic isomers. For each compound, the lowest electronic energy isomer was chosen as zero for the energy scale. Relative Gibbs’ energy values, estimated via the harmonic oscillator approximation, are also provided (between round brackets); the negative values found for TS geometries are due to shortcomings in the harmonic oscillator approximation.

Equilibrium geometries for the enolic tautomers of species **1**, **2**, and **3** in the ground state (GS), as well for the same tautomers of species **2** and **3** in the first excited state (ES), were optimized as indicated in Section 2. Figure 10 shows the final structure for **1**, **2**, and **3** when in the GS; the structures obtained for **2** and **3** in the ES are quite similar to the one in the GS and are, thus, not shown. De facto, the major difference between the equilibrium geometries in the two electronic states is related to the slight contraction of the enolic O-H bond length upon electronic excitation (from 1.019 to 1.013 Å for **2**, and from 1.018 to 1.010 Å for **3**). Electronic and Gibbs energies relative to the lowest energy species are also provided alongside the structural details. Notice that two isomers are shown for both **2** and **3** as the latter are asymmetric. As for the relative Gibbs energy ordering of the latter, it is invariably found that the most stable isomers have the proton involved in the tautomeric process localized on the side of the phenyl ring whose substituents have been modified; however, in all cases, the energy difference between the two enolic isomers is extremely small, and the two species ought to have quite similar populations. Similar conclusions can be reached by comparing relative electronic energies. Local minimum energy structures were also optimized for diketo tautomers, with the results invariably suggesting the enolic forms to be, at least, 5 kcal/mol (21 kJ/mol) more stable than any of the diketo isomers.

The transition state (TS) geometries for the proton transfer between the enolic tautomers were also located and are shown in Figure 10, together with their relative energetics with respect to the lowest electronic/Gibbs energy species. The electronic energy barriers appear fairly low, spanning the range 1.5–1.6 kcal/mol (6.3–6.6 kJ/mol), compared with the room temperature thermal energy (i.e., 0.6 kcal/mol or 2.5 kJ/mol). This finding clearly indicates that the enolic proton ought to be considered fluxional and mainly delocalized. De facto, the relative Gibbs energies for the TS fully support this idea, as they assume either very small or even negative values. The latter finding is, obviously, a consequence of the harmonic oscillator approximation used to estimate the vibrational partition function for isomers and TSs, which may display shortcomings when barriers are low; TS geometries are quite similar to local minima, and, hence, anharmonicity may play a key role when light atoms/particles are involved in the transformations. This notwithstanding, our energetic results suggest interesting peculiarities in the proton transfer processes involving the two enolic isomers of **2** and **3**. In particular, one is led to conclude that the dynamically averaged position of the enolic proton ought to lie symmetrically between the two oxygen atoms involved in the proton transfer in the case of both species.

Substantially similar conclusions can be reached for the proton transfer process involving **2** and **3** in their first ES. Thus, the electronic energy results suggest that the proton is preferentially located, as in the GS structures, the energy differences between the enolic tautomers are quite small. Our fitting of the ES energies obtained during the relaxed scans along the hydroxyl bond length involved in the proton transfer suggests only small changes in height compared to the GS situation (1.5 and 0.8 kcal/mol). Consequently, the enolic proton should still be considered fluxional in all cases.

To support the analysis and rationalization of the results for the ES dynamics, which suggested the long time component of the ES depopulation process to be ascribable to solvent rearrangement-mediated ESIPT, and that the relative times scales deduced for **1**, **2**, and **3** may possibly be correlated with differences in relative acidity between the species, we also estimated the relative energetics involved during the proton exchange between the most stable enolic form of a species with the (anionic) conjugate base of another. Choosing **1** as a common reference, transferring a proton to the conjugate base of **2** and **3** involves, respectively, a change in the standard Gibbs energy of 0.16 and 1.33 kcal/mol. The latter results, hence, appear in good agreement with what was deduced based only on the decay times.

### 2.5. Estimation of Singlet Oxygen-Generation Efficiency

**Figure 11:**
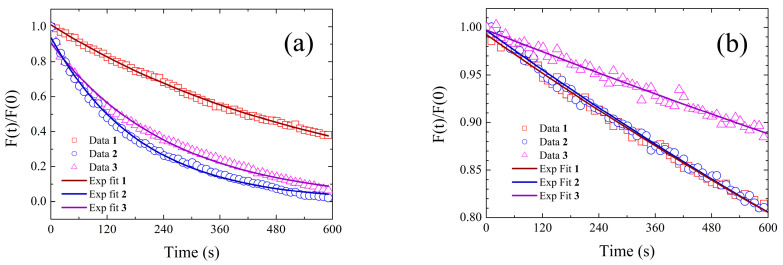
DMA fluorescence intensity (normalized to the zero-time value) as a function of irradiation time for solutions of DMA added with **1** (red squares), **2** (blue circles), and **3** (magenta triangles) in (**a**) toluene and (**b**) acetonitrile. The solid lines represent the best fits to a single exponential decay model function. The effects of DMA intrinsic fluorescence fading were taken into account as detailed in the Materials and Methods section.

In Figure 11, plots of the ratios between the DMA fluorescence drops in time measured in the presence of **1** (red squares), **2** (blue dots), and **3** (magenta triangles) and that measured in the absence of photosensitizer are shown. In both toluene (panel (a)) and acetonitrile (panel (b)), the presence of any of the tested curcuminoids in solution induces a DMA fluorescence that decreases over time more steeply than that measured in their absence. We ascribe this behavior to an increase in the DMA oxidation rate due to photosensitized ^1^O_2_ production. Accordingly, the fluorescence versus time patterns were fitted to single exponential decay functions, and the retrieved decay constants were assumed to be proportional to the ^1^O_2_ generation rates of the three tentative photosensitizers. Their values are listed in Table 11, whereby the ^1^O_2_ generation rates relative to **1** in the same solvent are also reported and compared to the corresponding *τ_av_* ratios.

Although it is worth recalling here that ^1^O_2_ generation is not believed to be the main photosensitizing mechanism for curcuminoids, the relative ^1^O_2_ generation rates roughly scale with the relative *τ_av_*, suggesting that the stabilization of the excited state is indeed a relevant strategy to pursue optimized photosensitized activity for this class of pharmaceutical active principles. The slight discrepancies between the relative ^1^O_2_ generation rates and the corresponding *τ_av_* ratios may be ascribed to differences in the curcuminoids’ photodegradation rates. Indeed, our control experiments (see Materials and Methods for details) unraveled that, among the three tested curcuminoids, **1** is the most photolabile in toluene, while **3** is the most photolabile in acetonitrile (data not shown). Most importantly, the scaling only holds once the environment in which the photosensitizing reaction is to occur is fixed, indicating that the environmental conditions are crucial in establishing the extent of production of the reactive species. Accordingly, although the excited-state lifetime is longer in the polar environment, the ^1^O_2_ generation appears to be fostered by a non-polar reaction medium.

## 3. Materials and Methods

*Chemicals and samples preparation.* Because the significant content of demethoxy and bis-demethoxycurcumin was reported for commercial curcumin samples, pure **1** was synthesized as described in [60]. The asymmetric compounds **2** and **3** were synthesized according to the procedure described in [51]. In the quoted manuscript, a full NMR characterization of the compounds was also performed. The solvents used for UV-Vis absorption and fluorescence experiments were supplied by Merck. They were ≥99.5% pure and were used as received. In Table 1, the values of dielectric constant, ε, and Kamlet–Taft acidity parameter, α, and basicity parameter, β [61], of the utilized solvents are reported. The NMR spectra were measured in toluene-*d*_8_, chloroform-*d*_1_, dichloromethane-*d*_2_, acetonitrile-*d*_3_, and dimethylsulfoxide-*d*_6_. Deuterated solvents were provided by Merck.

*Steady-state spectroscopy.* The UV-Vis absorption spectra were recorded with a Perkin Elmer Lambda 2 spectrophotometer. The molar extinction coefficients were determined through linear fit of absorbance versus concentration plots. The latter were obtained by measuring the spectra of at least five solutions of different concentrations for each solvent. To this end, a stock solution with concentrations in the millimolar range was prepared by weight, and suitably diluted samples with peak absorbance in the range 0.1–1 were obtained using precision micro-pipettes and cross-checking the volumes by weight.

The fluorescence emission and excitation spectra were measured with a PTI Fluorescence Master System fluorimeter. This was interfaced with the acquisition software Felix2000, which performed real-time spectra correction with respect to the excitation lamp spectral radiance and the detector quantum efficiency. Fluorescence quantum yields, ϕ_Fl_, were estimated relative to a fluorescence standard, namely, a solution of dimethyl-POPOP in cyclohexane (ϕ_Fl_ = 0.95) [62]. In so doing, the measured integral fluorescence intensities were suitably normalized to the relative absorbance of the specimens at the pertaining excitation wavelengths, and differences in the solvent’s refractive index were taken into account [48]. The ϕ_Fl_ values reported hereafter are averaged over three parallel samples, with errors given by the pertaining standard deviation.

The NMR measurements were performed with a Bruker ADVANCE 400 spectrometer. Then, ^1^H and ^13^C spectra were acquired at 400 MHz and 100 MHz, respectively, at room temperature (T = 298 K), with pulse delay 10 s, on 1.5 10^−2^ M solutions of **1**, **2,** and **3** in the deuterated solvents quoted above.

*Time-resolved fluorescence.* The fluorescence decay patterns were reconstructed by means of a TCSPC apparatus endowed with <30 ps temporal resolution, which is described in more detail in [63]. Briefly, the fluorescence of the solutions was excited by means of the built-in second harmonic (420 nm wavelength) of a SESAM mode-locked Ti:Sapphire laser (Tiger ps SHG, Time Bandwidth Products, Zurich, Switzerland) and detected through a 450 nm long-wavelength pass filter (Corion, Holliston, MA, USA) by means of an SPCM single-photon avalanche diode (MicroPhoton Devices, Bolzano, Italy). The detection times of fluorescence photons relative to the excitation laser pulses were determined, digitized, and sampled by an integrated time-correlated single-photon counting (TCSPC) PC board (SPC150, Becker & Hickl GmbH, Berlin, Germany). The so-obtained fluorescence decay distributions were fitted to multi-exponential decay functions over a constant background, applying the Levenberg–Marquardt χ^2^ minimization algorithm implemented within the data analysis software Origin 7. For each decay pattern, the number of decay components was determined by adding components one by one until further addition resulted in the determination of more than one component with the same decay constant. The decay times and relative amplitudes reported in the Results and Discussion section are the averages of the values obtained from the fits of six measured decay patterns, with errors expressed in terms of the corresponding standard deviations.

*Spectrofluorimetric detection of photosensitized ROS generation.* The relative efficiency of **2** and **3** with respect to **1** in photosensitizing singlet oxygen (^1^O_2_) generation was assessed in toluene and acetonitrile by exploiting the DMA fluorescence assay [64]. Due to its proneness to oxidation, a fresh 1 mM concentrated stock of DMA was prepared from the powdered compound immediately before each singlet oxygen-generation measurement. Optimal solubilization was pursued by stirring the stock with a magnetic anchor for 30 min. During this procedure, the sample was kept in the dark in sealed vials after air removal through nitrogen flux. Then, 1 µM concentrated solutions of the three compounds were prepared by weighing suitable amounts of the powders. The concentration was cross-checked spectrophotometrically, using the molar extinction coefficient values determined as described above, and matched the nominal value within the experimental errors of the molar extinction coefficients. DMA was added from the 1 mM stocks at a final concentration of 10 μM. The obtained samples were placed in 1 × 1 cm^2^ fluorimetry quartz cells to carry out the fluorescence measurements.

The ^1^O_2_ generation measurements were performed using the PTI fluorimeter to convey the suitable light dose to the photosensitizers and simultaneously detect the probe response. The samples were illuminated in a 20 nm-wide band centered at 420 nm, setting the lamp power at 85 W. With these instrumental settings, the spectral radiance of the lamp in the selected band is comparable to that of sunlight. The DMA fluorescence was excited out-of-peak by the same light used to elicit photosensitization, and detected in real time for 10 min in a 2 nm-wide band around the 395 nm emission peak. Control measurements were carried out in the same experimental conditions on DMA alone. Since the DMA fluorescence was observed to decrease slightly over time, even in the absence of photosensitizers, likely due to a combination of residual oxidation and photobleaching, the data were corrected by dividing point-by-point the decays obtained in the presence of the photosensitizer by those obtained with the DMA alone, after the suitable normalization and baseline subtraction of each data string. Further control experiments, conducted on the photosensitizers in the absence of DMA, evidenced that photodegradation, although being significant for all three compounds on longer timescales, can be neglected over the 10 min acquisition time chosen for ^1^O_2_ generation estimation. For these studies, excitation was elicited with the same light dose (i.e., the same spectral band and lamp intensity), but the fluorescence of the photosensitizers was monitored over time in a 2 nm-wide band around their emission peaks.

*Theoretical modeling.* Electronic structure calculations were carried out employing the B3LYP/6-31++G(d, p) method for both ground and excited states. The latter were obtained using the Time-Dependent Density Function Theory approach, as implemented in the Gaussian09 suite of codes. The local geometry optimization for both ground and excited states started from suitable putative geometries; as for the former cases, these were obtained using a force field-based representation of the structural energies. Optimizations over the excited state surfaces were instead started using the local ground state minima as parent structures, as no major structural changes were expected upon excitation. Indeed, we verified a posteriori the correctness of such assumption. The transition state (TS) geometries for the enol proton transfer in both ground and excited states were initially located in an approximate way via relaxed scans along the oxygen–hydrogen bond length; given the availability of analytical second derivatives, the approximate ground-state TS geometries were further refined. Fitting with a second-degree polynomial was instead exploited to determine the TS energy and equilibrium distance for proton transfer in the excited state.

## 4. Conclusions

Curcuminoids are being increasingly investigated as photosensitizers. Their optimal exploitation is hindered by their very fast decay from the excited state. The propensity to undergo excited-state intramolecular proton transfer of the enol proton has a major role in such instability. In this article, we tested the benefits induced by the asymmetrization of the molecular structure on the inhibition of proton-transfer mechanisms by analyzing two synthetic phenyl-substituted curcumin analogs. Although the excited-state dynamics have shown to be dependent on the specific aromatic ring substituents chosen to pursue asymmetrization, our results demonstrate that for both asymmetric curcuminoids, the excited-state proton transfer is both slower and significantly less probable than for the corresponding symmetric curcuminoid, thus encouraging further synthetic efforts in this field.

## Figures and Tables

**Table 1:** Chemical–physical properties of the used solvents.

Environment	Solvent	Ε	α	β
Non-polar	Cyclohexane	2.02	0	0
	Toluene	2.38	0	0.12
Polar weakly H-bonding	Chloroform	4.81	0.44	0
	Dichloromethane	8.93	0.13	0.10
	Acetone	20.6	0.08	0.48
	Acetonitrile	38.8	0.19	0.31
H-bond acceptors	Dimethylformamide	37.6	0	0.69
	Dimethylsulfoxide	48.9	0	0.76
Alcohols	Butanol	17.51	0.79	0.88
	Ethanol	25.07	0.83	0.77
	Methanol	33.62	0.93	0.62
	Ethylene glycol	37.7	0.90	0.52

**Table 2:** Absorption peak wavelength of **2** in the different solvents of Table 1, λ_abs,**2**_, is compared to that of **1**, λ_abs,**1**_, reproduced from [38] for the sake of straightforward comparison. The molar extinction coefficient of **2** at the absorption peak, ε**_2_**, is also reported.

Solvent	λ_abs,2_	λ_abs,1_	ε_2_
Cyclohexane	409, (430)	408, 429	-
Toluene	421, (439)	(399), 417, (437) ^1^	30,765 ± 2400
Chloroform	421	419	35,400 ± 2700
Dichloromethane	421	418 ^1^	31,600 ± 2200
Acetone	421	420	33,000 ± 2400
Acetonitrile	421	419	35,300 ± 2100
Dimethylformamide	426	431	31,300 ± 1700
Dimethylsulfoxide	431	434	32,600 ± 1900
Butanol	421	431 ^1^	31,800 ± 2300
Ethanol	421	430	33,000 ± 2000
Methanol	421	423	38,600 ± 3200
Ethylene glycol	428	433	-

^1^ Not determined in [38], measured for this article.

**Table 3:** Emission peak wavelength, λ_emi,**2**_, and the fluorescence quantum yield, *ϕ_fluo_*_,**2**_, of **2** in the different solvents of Table 1 are compared to those of **1**, λ_emi,**1**_ and *ϕ_fluo_*_,**1**_, reproduced from [38] for the sake of straightforward comparison. The peak excitation wavelength of **2**, λ_exc,**2**_, is also reported.

Solvent	λ_emi,2_	λ_emi,1_	*ϕ_fluo_*_,2_	*ϕ_fluo_*_,1_	λ_exc,2_
Cyclohexane	472 (500)	443, 471, 502	-	0.006 ± 0.001	412 (434)
Toluene	461, 489	459, 487 ^1^	0.47 ± 0.02	0.036 ± 0.001 ^1^	421 (443)
Chloroform	482	503	0.50 ± 0.03	0.094 ± 0.005	422
Dichloromethane	488	495 ^1^	0.62 ± 0.03	0.189 ± 0.007 ^1^	421
Acetone	497	510	0.58 ± 0.03	0.174 ± 0.006	421
Acetonitrile	506	521	0.41 ± 0.02	0.156 ± 0.003	421
Dimethylformamide	521	536	0.11 ± 0.01	0.041 ± 0.001	434
Dimethylsulfoxide	522	550	0.13 ± 0.01	0.026 ± 0.002	433
Butanol	526	540 ^1^	0.23 ± 0.01	0.104 ± 0.003 ^1^	431
Ethanol	531	553	0.16 ± 0.02	0.033 ± 0.004	423
Methanol	535	566	0.064 ± 0.007	0.028 ± 0.002	426
Ethylene glycol	535	566	-	0.022 ± 0.004	435

^1^ Not determined in [38], measured for this article.

**Table 4:** Decay times, *τ_i_*, and pertaining relative amplitudes resolved in the fluorescence decay distributions measured for **2** in the different solvents of Table 1. In the last two columns the corresponding average S_1_ state lifetimes, *τ_av_*_,**2**_, calculated according to Equation (1), are compared to those of **1**, *τ_av_*_,**1**_, reproduced from [38] for the sake of straightforward comparison.

Solvent	*τ*_1_ (ps) (f_1_)	*τ*_2_ (ps) (f_2_)	*τ*_3_ (ps) (f_3_)	*τ_av_*_,2_ (ps)	*τ_av_*_,1_ (ps)
Cyclohexane	41 ± 1 (0.36)	204 ± 1 (0.63)	1413 ± 55 (<0.01)	151 ± 10	89
Toluene	277 ± 4			277 ± 4	106 ^1^
Chloroform	432 ± 6			432 ± 6	596
Dichloromethane	544 ± 1			544 ± 1	483 ^2^
Acetone	665 ± 3			665 ± 3	702
Acetonitrile	759 ± 1			759 ± 1	695
Dimethylformamide	230 ± 2 (0.98)	1115 ± 88 (0.02)		248 ± 6	251
Dimethylsulfoxide	243 ± 1 (≈1)	1581 ± 46 (<0.01)		244 ± 3	155
Butanol	412 ± 2	1630 ± 140 (<0.01)		416 ± 2	527 ^2^
Ethanol	277 ± 5 (≈1)	1275 ± 25 (<0.01)		278 ± 6	260
Methanol	182 ± 3 (0.87)	350 ± 18 (0.13)		204 ± 10	162
Ethylene glycol	257 ± 2	1950 ± 190 (<0.01)		259 ± 2	232

^1^ Not determined in [38], measured for this article (see text for details on fitting parameters). ^2^ Not determined in [38], measured for this article (single exponential decay).

**Table 5:** Radiative and non-radiative decay rates, *k_fluo_*_,**2**_ and *k_nr_*_,**2**_, calculated for **2** in the different solvents of Table 1, are compared to those of **1**, *k_fluo_*_,**1**_ and *k_nr_*_,**1**_, reproduced from [38].

Solvent	*k_fl_*_,2_ (s^−1^)	*k_fl_*_,1_ (s^−1^)	*k_nr_*_,2_ (s^−1^)	*k_nr_*_,1_ (s^−1^)
Cyclohexane	-	6.7 × 10^7^		1.12 × 10^10^
Toluene	(1.7 ± 0.1) × 10^9^	3.4 × 10^8^ *	(1.9 ± 0.1) × 10^9^	9.09 × 10^9^ *
Chloroform	(1.16 ± 0.09) × 10^9^	1.6 × 10^8^	(1.2 ± 0.1) × 10^9^	1.51 × 10^9^
Dichloromethane	(1.14 ± 0.06) × 10^9^	1.97 × 10^8^ *	(7.0 ± 0.6) × 10^8^	1.70 × 10^9^ *
Acetone	(8.7 ± 0.5) × 10^8^	2.5 × 10^8^	(6.3 ± 0.6) × 10^8^	1.42 × 10^9^
Acetonitrile	(5.4 ± 0.3) × 10^8^	2.2 × 10^8^	(7.8 ± 0.3) × 10^8^	1.22 × 10^9^
Dimethylformamide	(4.4 ± 0.5) × 10^8^	1.6 × 10^8^	(3.6 ± 0.1) × 10^9^	3.82 × 10^9^
Dimethylsulfoxide	(5.3 ± 0.5) × 10^8^	1.7 × 10^8^	(3.6 ± 0.1) × 10^9^	6.28 × 10^9^
Butanol	(5.5 ± 0.3) × 10^8^	3.9 × 10^8^ *	(1.85 ± 0.04) × 10^9^	1.68 × 10^9^ *
Ethanol	(5.8 ± 0.8) × 10^8^	1.3 × 10^8^	(3.0 ± 0.2) × 10^9^	3.72 × 10^9^
Methanol	(3.1 ± 0.5) × 10^8^	1.7 × 10^8^	(4.6 ± 0.3) × 10^9^	6.00 × 10^9^
Ethylene glycol	-	9.5 × 10^8^		3.36 × 10^9^

* Not determined in [38], measured for this article.

**Table 6:** Absorption peak wavelength of **3** in the different solvents of Table 1, λ_abs,**3**_, is compared to that of DCMeth, λ_abs,DCMeth_, reproduced, when available, from [39] for the sake of straightforward comparison. The molar extinction coefficient of **3** at the absorption peak, ε**_3_**, is also reported.

Solvent	λ_abs,3_	λ_abs,DCMeth_	ε_3_
Cyclohexane	402 (428)	388	25,800 ± 2300
Toluene	409 (431)	-	22,900 ± 3400
Chloroform	405	395	26,500 ± 2200
Dichloromethane	408	-	30,100 ± 2300
Acetone	409	391	30,400 ± 2100
Acetonitrile	406	390	27,300 ± 2400
Dimethylformamide	417	397	30,100 ± 1800
Dimethylsulfoxide	421	399	28,000 ± 2300
Butanol	415	-	30,800 ± 2100
Ethanol	412	393	34,000 ± 1900
Methanol	412	391	29,800 ± 3000
Ethylene glycol	418	-	34,000 ± 2700

**Table 7:** Emission peak wavelength, λ_emi,**3**_, and the fluorescence quantum yield, *ϕ_fluo_*_,**3**_, of **3** in the different solvents of Table 1 are compared to those of DCMeth, λ_emi,DCMeth_ and *ϕ_fluo_*_,DCMeth_, reproduced from [39] for the sake of straightforward comparison. The peak excitation wavelength of **3**, λ_exc,**3**_, is also reported.

Solvent	λ_emi,3_	λ_emi,DCMeth_	*ϕ_fluo_*_,3_	*ϕ_fluo_*_,DCMeth_	λ_exc,3_
Cyclohexane	438, 464 (493)	427, 449, 472	0.025 ± 0.001	0.0008 ± 0.0001	403 (425)
Toluene	459 (479)	-	0.132 ± 0.001	-	413 (430)
Chloroform	490	435, 462	0.160 ± 0.002	0.0026 ± 0.0001	412
Dichloromethane	492	-	0.307 ± 0.003	-	411
Acetone	515	428, 459	0.137 ± 0.002	0.0018 ± 0.0001	413
Acetonitrile	521	434, 457	0.183 ± 0.002	0.0017 ± 0.0001	410
Dimethylformamide	538	437, 463	0.009 ± 0.001	0.0024 ± 0.0001	421
Dimethylsulfoxide	544	441, 469	0.011 ± 0.001	0.0060 ± 0.0004	422
Butanol	533	-	0.040 ± 0.001	-	420
Ethanol	532	441, 461	0.031 ± 0.001	0.0040 ± 0.0003	418
Methanol	533	459, 481	0.012 ± 0.001	0.0020 ± 0.0003	416
Ethylene glycol	533	-	0.030 ± 0.001	0.022 ± 0.004	421

**Table 8:** Decay times, *τ_i_*, and pertaining relative amplitudes resolved in the fluorescence decay distributions measured for **3** in the different solvents of Table 1. In the last two columns, the corresponding average S_1_ state lifetimes, *τ_av_*_,**3**_, calculated according to Equation (1), are compared to those of DCMeth, *τ_av_*_,DCMeth_, reproduced from [39] for the sake of straightforward comparison.

Solvent	*τ*_1_ (ps) (f_1_)	*τ*_2_ (ps) (f_2_)	*τ*_3_ (ps) (f_3_)	*τ_av_*_,3_ (ps)	*τ_av_*_,DCMeth_ (ps)
Cyclohexane	26 ± 1 (0.74)	244 ± 3 (0.26)	2400 ± 90 (<0.01)	84 ± 3	104
Toluene	208 ± 1 (≈1)	1105 ± 83 (<0.01)		210 ± 2	-
Chloroform	306 ± 1 (0.97)	1541 ± 5 (0.03)		338 ± 1	91
Dichloromethane	446 ± 2 (0.98)	1030 ± 55 (0.02)		458 ± 1	-
Acetone	572 ± 1			572 ± 1	211
Acetonitrile	515 ± 1			515 ± 1	117
Dimethylformamide	47 ± 1 (0.67)	201 ± 2 (0.33)	1030 ± 24 (<0.01)	97 ± 1	232
Dimethylsulfoxide	57 ± 1 (0.62)	210 ± 1 (0.38)	1660 ± 33 (<0.01)	114 ± 1	101
Butanol	287 ± 2			287 ± 2	-
Ethanol	205 ± 1			205 ± 1	178
Methanol	54 ± 1 (0.66)	221 ± 1 (0.34)		112 ± 1	602
Ethylene glycol	93 ± 3 (0.39)	226 ± 1 (0.61)		174 ± 2	-

**Table 9:** Radiative decay rates, *k_fluo_*_,**3**_, and non-radiative decay rates, *k_nr_*_,**3**_, calculated for **3** in the different solvents of Table 1 according to Equations (2) and (3) are compared to those of DCMeth, *k_fluo_*_,DCMeth_ and *k_nr_*_,DCMeth_, reproduced from [39] for the sake of straightforward comparison.

Solvent	*k_fl_*_,3_ (s^−1^)	*k_fl_*_,DCMeth_ (s^−1^)	*k_nr_*_,3_ (s^−1^)	*k_nr_*_,1_ (s^−1^)
Cyclohexane	(3.0 ± 0.1) × 10^8^	8 × 10^6^	(1.16 ± 0.4) × 10^10^	9.61 × 10^9^
Toluene	(6.3 ± 0.1) × 10^8^	-	(4.13 ± 0.06) × 10^9^	-
Chloroform	(4.72 ± 0.07) × 10^8^	2.8 × 10^7^	(2.49 ± 0.03) × 10^9^	1.096 × 10^10^
Dichloromethane	(9.1 ± 0.1) × 10^8^	-	(2.05 ± 0.02) × 10^9^	-
Acetone	(3.00 ± 0.04) × 10^8^	9 × 10^6^	(1.88 ± 0.01) × 10^9^	4.74 × 10^9^
Acetonitrile	(3.22 ± 0.04) × 10^8^	1.5 × 10^7^	(1.43 ± 0.01) × 10^9^	8.53 × 10^9^
Dimethylformamide	(1.84 ± 0.02) × 10^7^	1.0 × 10^7^	(1.92 ± 0.02) ×10^9^	4.31 × 10^9^
Dimethylsulfoxide	(1.15 ± 0.02) × 10^8^	6.0 × 10^7^	(10.2 ± 0.1) × 10^9^	9.93 × 10^9^
Butanol	(3.47 ± 0.07) × 10^8^	-	(8.42 ± 0.08) × 10^9^	-
Ethanol	(1.04 ± 0.02) × 10^8^	2.2 × 10^7^	(3.39 ± 0.01) × 10^9^	5.60 × 10^9^
Methanol	(1.51 ± 0.03) × 10^8^	3 × 10^6^	(4.73 ± 0.05) × 10^9^	1.66 × 10^9^
Ethylene glycol	(1.04 ± 0.02) × 10^8^	-	(8.82 ± 0.08) × 10^9^	-

**Table 10:** Value of the A parameter estimated for **1**, **2**, and **3** using Abraham’s empirical formula.

Species 1	Species 2	Species 3
δ_OH enol_(DMSO) = 16.50 ppm δ_OH enol_(CDCl_3_) = 16.33 ppm	δ_OH enol_(DMSO) = 16.40 ppm δ_OH enol_(CDCl_3_) = 16.07 ppm	δ_OH enol_(DMSO) = 16.30 ppm δ_OH enol_(CDCl_3_) = 15.99 ppm
**A = 0.029**	**A = 0.033**	**A = 0.048**

**Table 11:** DMA fluorescence fading decay constants, obtained by fitting the data of Figure 11 (column three), are used to extract estimates of the relative ^1^O_2_ generation quantum yields (column four), which are compared with the relative excited-state stabilities, measured in terms of ratios (column five).

Solvent	Photosensitizer	k_i_ (10^−3^s^−1^)	k(^1^O_2_)/k(^1^O_2_)_1_	*τ*_*av,i*_/*τ*_*av*,1_
	**1**	0.348	1.00	1
Acetonitrile	**2**	0.355	1.02	1.09
	**3**	0.193	0.56	0.74
	**1**	1.67	1	1
Toluene	**2**	5.26	3.15	2.61
	**3**	3.92	2.35	1.98

## Data Availability

Data is contained within the article and Supplementary Material.

## Supplementary Materials

The following supporting information can be downloaded at: https://www.mdpi.com/article/10.3390/ph15070843/s1, Figure S1. 1H-NMR spectra of **2** in selected solvents. From top to bottom: Toluene-*d*_8_; Chloroform-*d*, Dichloromethane-*d*_2_, and Acetonitrile-*d*_3_; Figure S2. ^1^H-NMR spectra of **3** in selected solvents. From top to bottom: Toluene-*d*_8_; Chloroform-*d*, Dichloromethane-*d*_2_, and Acetonitrile-*d*_3_; Figure S3. ^1^H-NMR spectra of **2** in Toluene-*d_8_* at selected temperatures from room temperature to 188 K; Figure S4. ^1^H-NMR spectra of **3** in Toluene-*d*_8_ at selected temperatures from room temperature to 188 K; Figure S5. Zoom on the enolic proton region of the ^1^H-NMR spectra of **2** in Toluene-*d*_8_ at selected temperature ranging from room temperature to 188 K; Figure S6. Zoom on the enolic proton region of the ^1^H-NMR spectra of **3** in Toluene-*d*_8_ at selected temperature ranging from room temperature to 188 K.
