# Supplementary material for: Asymmetric Phenyl Substitution: An Effective Strategy to Enhance the Photosensitizing Potential of Curcuminoids
Source: Pharmaceuticals (Basel). 2022 Jul 9;15(7):843. doi: 10.3390/ph15070843 (PMC9321223; doi:10.3390/ph15070843)
Supplement: Supplementary file 1 [file pharmaceuticals-15-00843-s001.zip › pharmaceuticals-1772393-supplementary.pdf]

pharmaceuticals

Supplementary Online Materials

Asymmetric Phenyl Substitution: An Effective Strategy to Enhance the Photosensitizing Potential of Curcuminoids

Guglielmo Vesco [1,†], Martino Brambati [1,†], Luca Scapinello [1], Andrea Penoni [1], Massimo Mella [1], Màr Masson [2], Vivek Gaware[2], Angelo Maspero [1,*], Luca Nardo [1,*]

[1] Università degli Studi dell'Insubria – Dept. of Science and High Technology, Via Valleggio 11, 22100 Como (CO); gvesco@uninsubria.it (G.V.); mbrambati1@studenti.uninsubria.it (M.B.); l.scapinello@uninsubria.it (L.S.); andrea.penoni@uninsubria.it (A.P.); massimo.mella@uninsubria.it (M.M.); angelo.maspero@uninsubria.it (A.M.); luca.nardo@uninsubria.it (L.N.)
[2] University of Iceland – School of Health Sciences, Saemundargata 2, 102 Reykjavìk; mmasson@hi.is
[†] These authors contributed equally
[*] Correspondence: luca.nardo@uninsubria.it ; angelo.maspero@uninsubria.it

Citation: Vesco, G.; Brambati, M.; Scapinello, L.; Penoni, A.; Mella, M.; Masson, M.; Gaware, V.; Maspero, A.; Nardo, L. Asymmetric Phenyl Substitution: An Effective Strategy to Enhance the Photosensitizing Potential of Curcuminoids. *Pharmaceuticals* **2022**, *15*, 843. https://doi.org/10.3390/ph15070843

Academic Editor: Francois Dufrasne

Received: 30 May 2022
Accepted: 6 July 2022
Published: 9 July 2022

Publisher's Note: MDPI stays neutral with regard to jurisdictional claims in published maps and institutional affiliations.

Copyright: © 2022 by the authors. Licensee MDPI, Basel, Switzerland. This article is an open access article distributed under the terms and conditions of the Creative Commons Attribution (CC BY) license (https://creativecommons.org/licenses/by/4.0/).

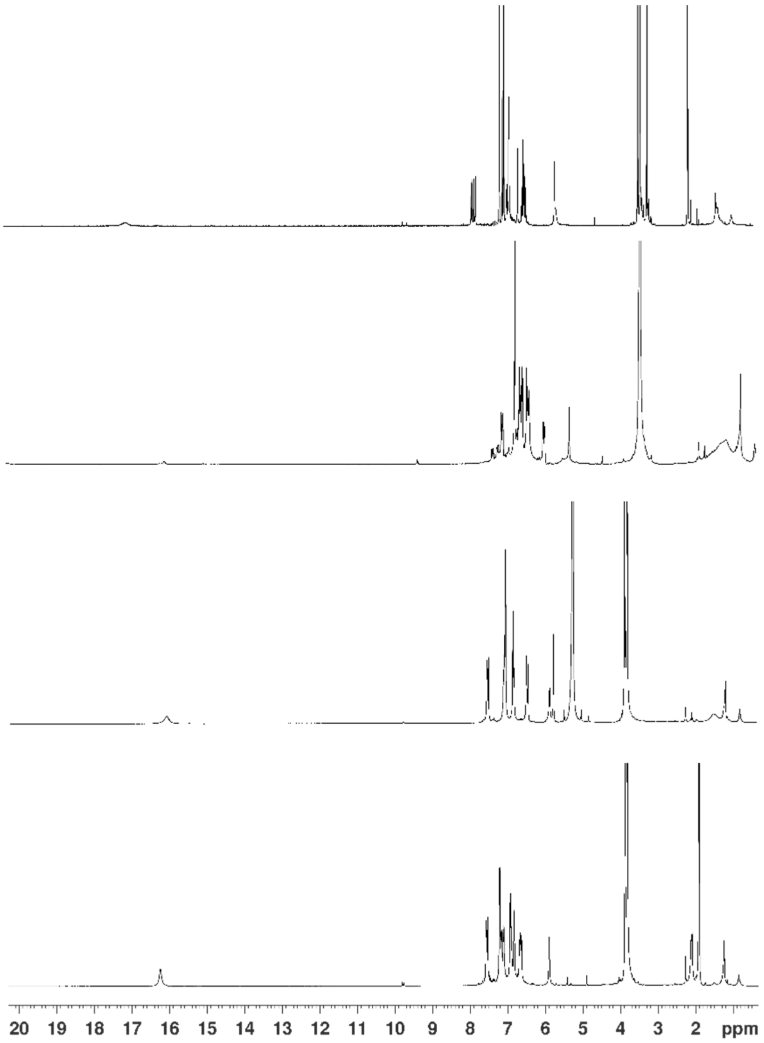

Figure S1. ^{1}H-NMR spectra of **2** in selected solvents. From top to bottom: Toluene-*d*$_8$; Chloroform-*d*, Dichloromethane-*d*$_2$, and Acetonitrile-*d*$_3$.

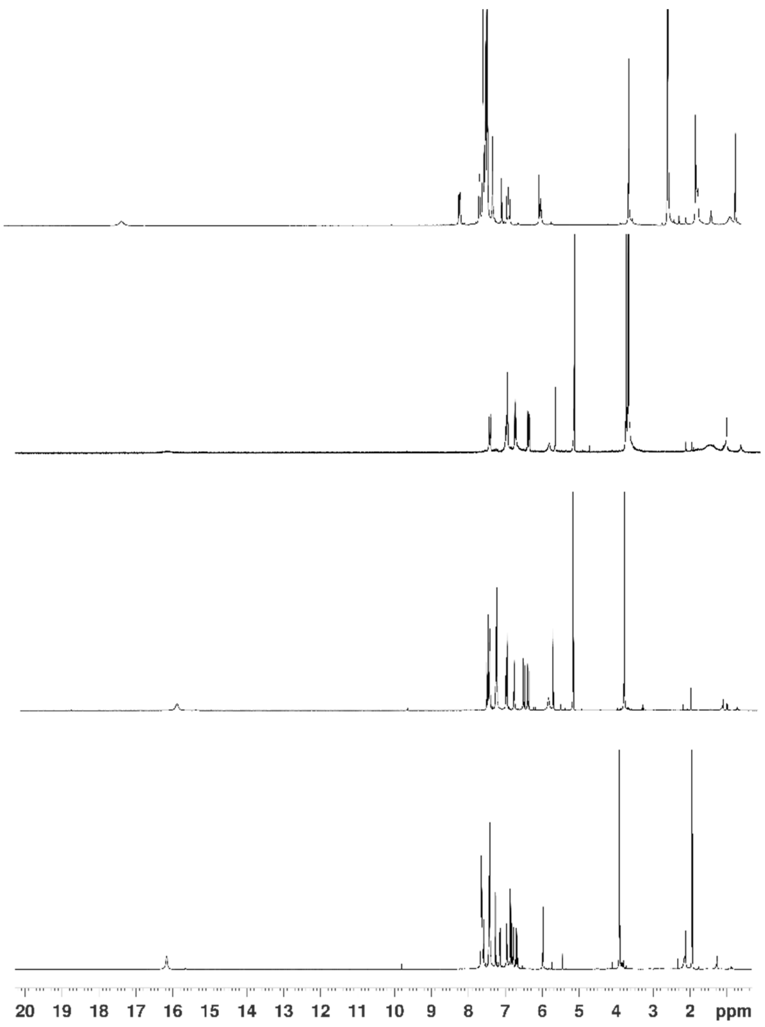

Figure S2. ^{1}H-NMR spectra of **3** in selected solvents. From top to bottom: Toluene-*d*$_8$; Chloroform-*d*, Dichloromethane-*d*$_2$, and Acetonitrile-*d*$_3$.

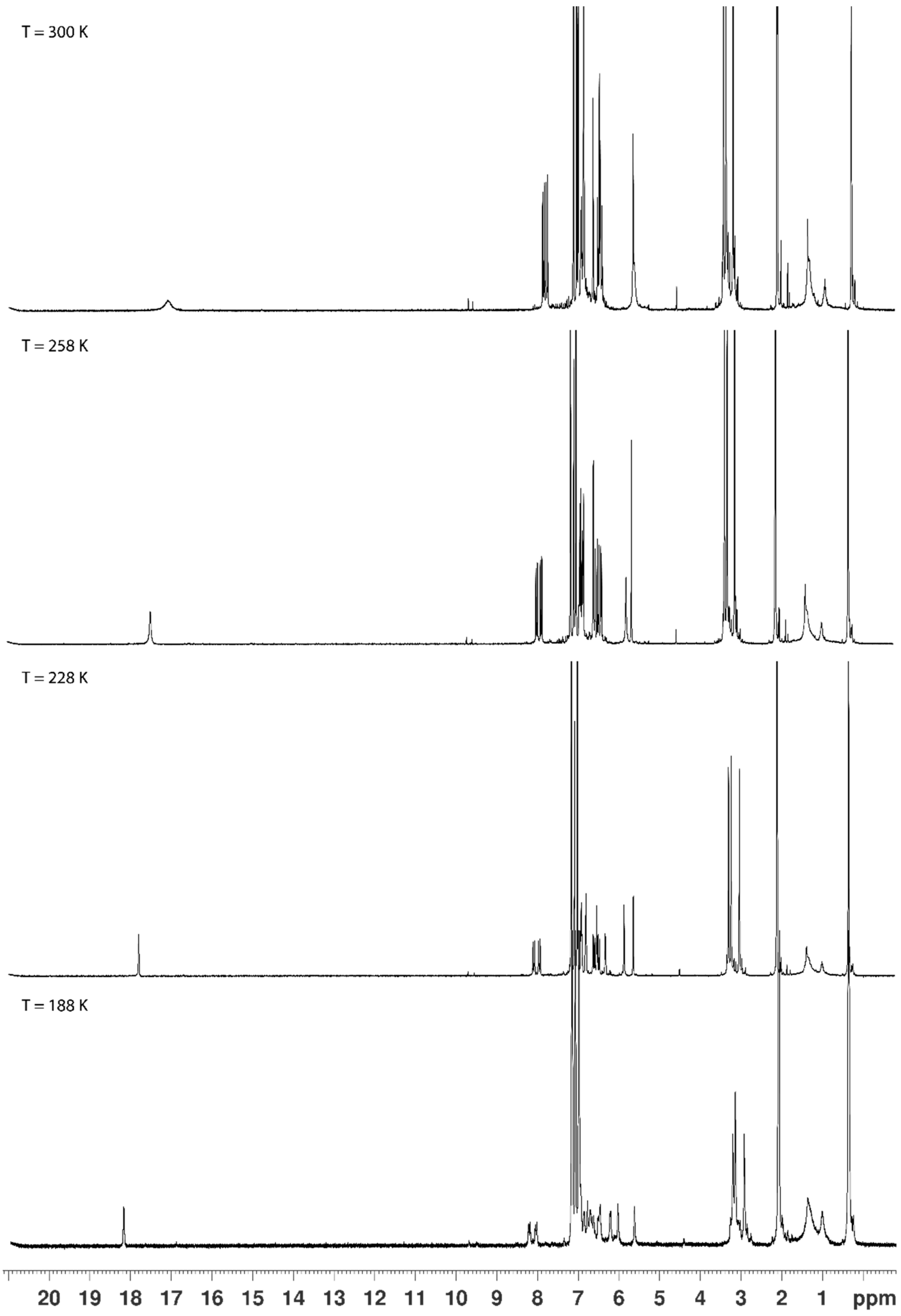

Figure S3. ^{1}H-NMR spectra of **2** in Toluene-d_8 at selected temperatures from room temperature to 188 K.

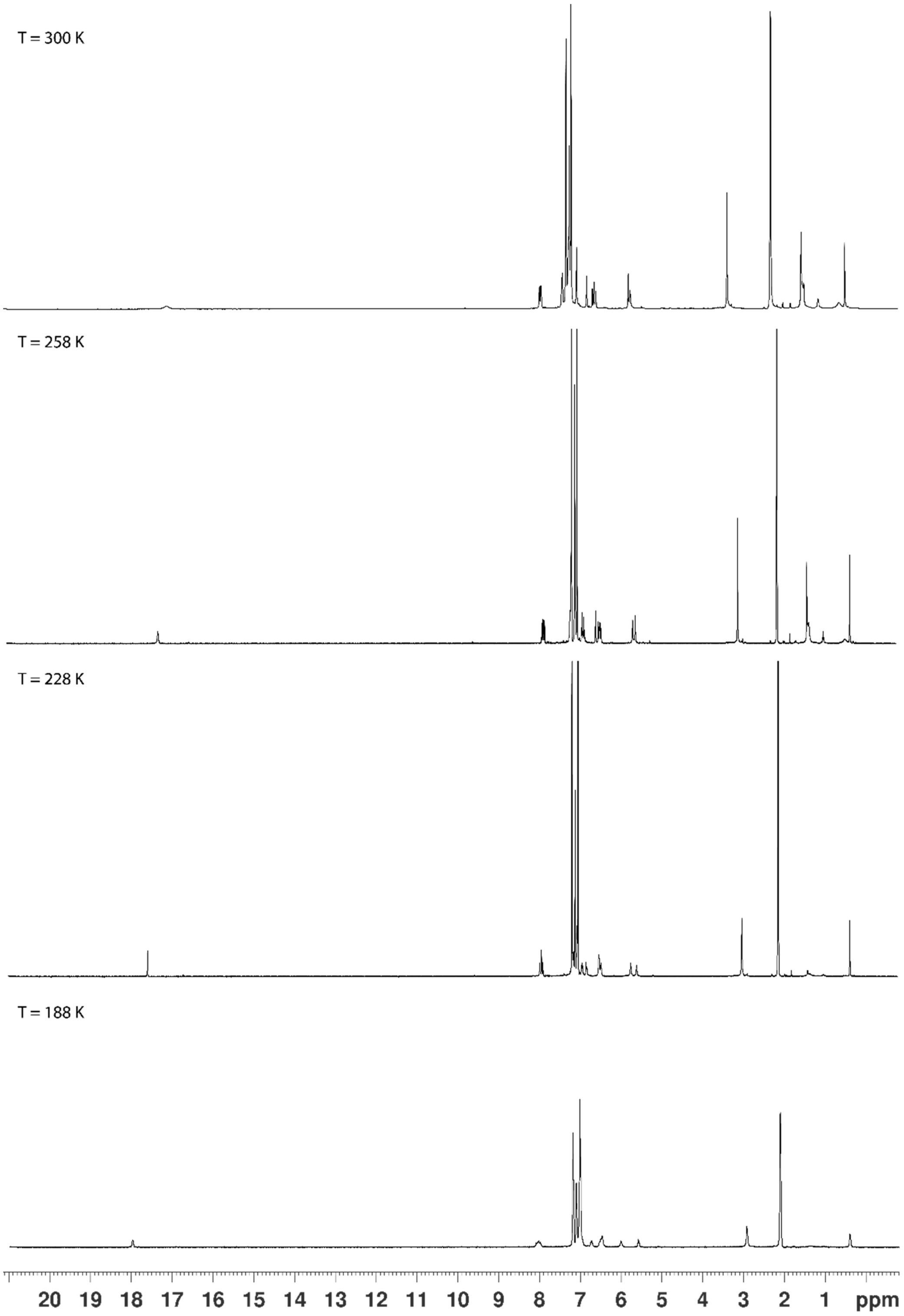

Figure S4. ^{1}H-NMR spectra of **3** in Toluene-*d8* at selected temperatures from room temperature to 188 K.

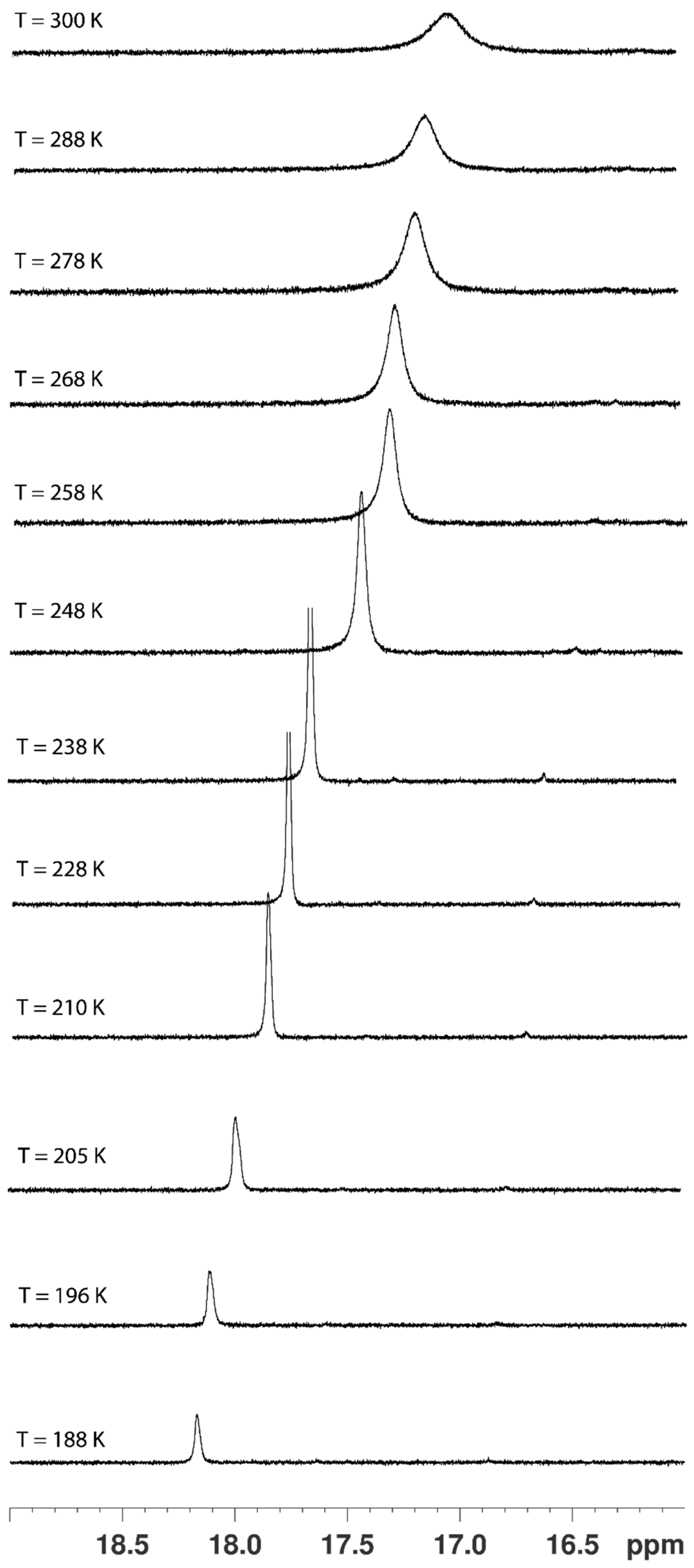

Figure S5. Zoom on the enolic proton region of the ^{1}H-NMR spectra of **2** in Toluene-d_8 at selected temperature ranging from room temperature to 188 K.

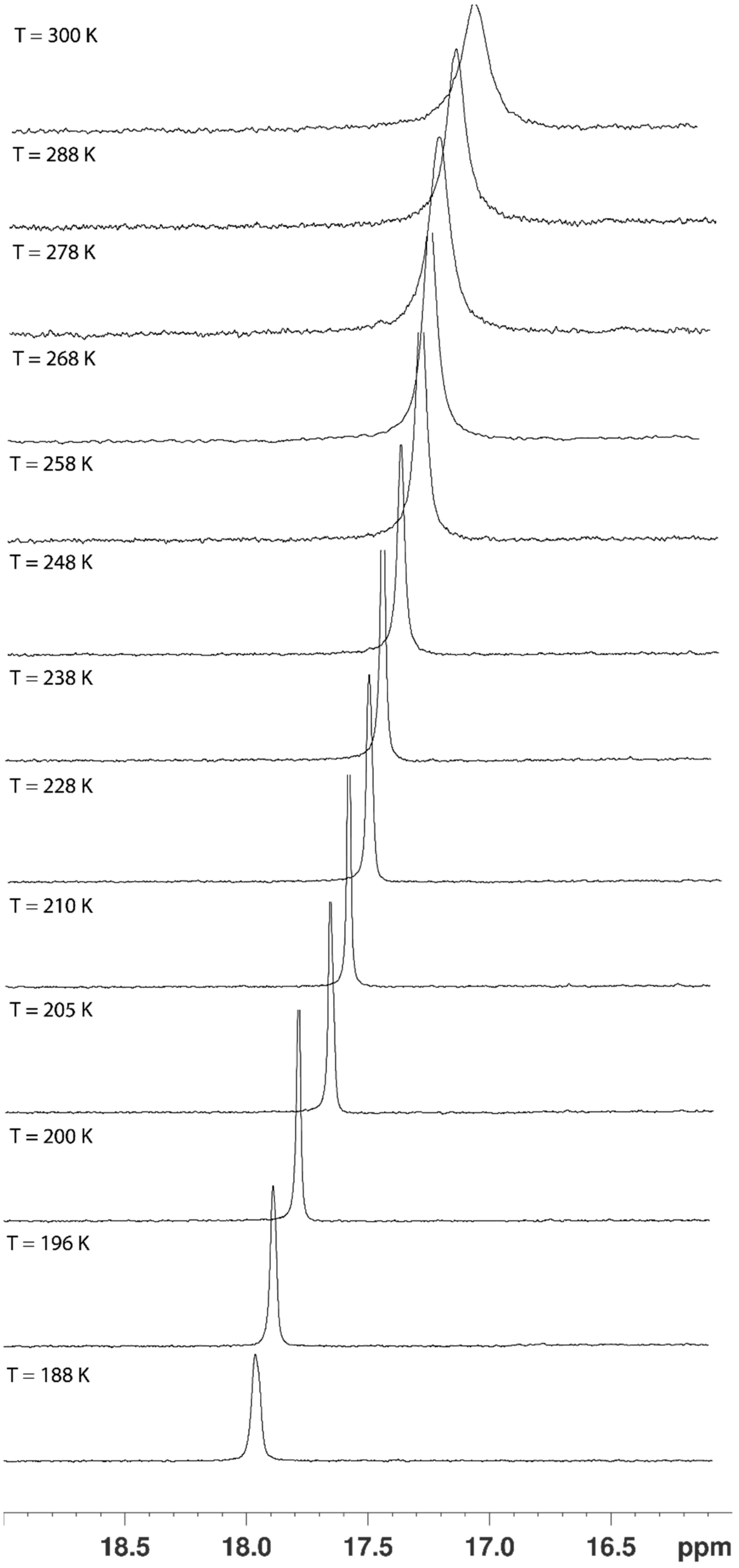

Figure S6. Zoom on the enolic proton region of the ^{1}H-NMR spectra of **3** in Toluene-d_8 at selected temperature ranging from room temperature to 188 K.
